# The Role of P Wave Parameters in Predicting Pulmonary Vein Isolation Outcomes for Paroxysmal Atrial Fibrillation: An Observational Cohort Study

**DOI:** 10.3390/jcdd11090277

**Published:** 2024-09-05

**Authors:** Ibrahim Antoun, Xin Li, Ahmed I. Kotb, Zakkariya Vali, Ahmed Abdelrazik, Abdulmalik Koya, Akash Mavilakandy, Ivelin Koev, Ali Nizam, Hany Eldeeb, Riyaz Somani, André Ng

**Affiliations:** 1 Department of Cardiology, University Hospitals of Leicester NHS Trust, Glenfield Hospital, Leicester LE3 9QP, UK; aiamk1@leicester.ac.uk (A.I.K.); z.vali@leicester.ac.uk (Z.V.); ahmed.abdelrazik@leicester.ac.uk (A.A.); aik8@leicester.ac.uk (A.K.); am1138@leicester.ac.uk (A.M.); ivelin.koev@uhl-tr.nhs.uk (I.K.); andre.ng@leicester.ac.uk (A.N.); 2 Department of Engineering, University of Leicester, Leicester LE1 7RH, UK; xin.li@leicester.ac.uk; 3 Department of Cardiovascular Sciences, Clinical Science Wing, University of Leicester, Glenfield Hospital, Leicester LE3 9QP, UK; ali.h.nizam@gmail.com (A.N.); hany.eldeeb@uhl-tr.nhs.uk (H.E.); 4 National Institute for Health Biomedical Centre, Leicester LE3 9QP, UK

**Keywords:** atrial fibrillation, P wave, P wave duration, P wave amplitude, catheter ablation

## Abstract

Background: Pulmonary vein isolation (PVI) is an effective management method for paroxysmal atrial fibrillation (PAF). The P wave in the 12-lead electrocardiogram (ECG) represents atrial depolarisation. This study aims to utilise the P wave to predict PVI outcomes for PAF. Methods: This single-centre retrospective study aimed to predict PVI outcomes using P wave parameters. It included 211 consecutive patients with first PVI for PAF between 2018 and 2019 and targeted the pulmonary veins (PVs). Procedure success was defined by freedom of ECG-documented AF at 12 months. Digital 12-lead ECGs with 1–50 hertz bandpass filters were monitored before the procedure. Corrected P wave duration (PWDc), P wave amplitude (PWV), P wave dispersion (PWDisp), intra-atrial block (IAB), P wave area (PWA), and P wave terminal force in V1 (PTFV1) were measured before ablation and correlated with the outcomes. Results: Successful PVI occurred in 154 patients (73%). Demographics were similar between both arms. P wave parameters correlated with PVI failure included increased PWDc in all leads except for lead III, aVR, and V3, decreased PWV in lead I (hazard ratio [HR]: 0.7, 95% confidence interval [CI]: 0.53–0.95), lead II (HR: 0.45, 95% CI: 0.22–0.65), aVL (HR: 0.58, 95% CI: 0.22–0.98), and aVF (HR: 0.67, 95% CI: 0.58–0.87), decreased PWA in lead I (HR: 0.55, 95% CI: 0.21–0.76), lead II (HR: 0.48, 95% CI: 0.34–0.87), aVL (HR: 0.65, 95% CI: 0.45–0.96), and aVF (HR: 0.61, 95% CI: 0.32–0.89), and the presence of IAB (HR: 2, 95% CI: 1.4–4.2, *p* = 0.02). PWDisp and PTFV1 were not correlated with PVI outcome. Conclusions: PWDc, PWA, PWV, and IAB are valuable predictors for PVI outcome for PAF at 12 months.

## 1. Introduction

AF ablation with the goal of pulmonary vein isolation (PVI) has emerged as an effective rhythm control method for paroxysmal atrial fibrillation (PAF) [1]. This is mainly conducted by electrically isolating the pulmonary veins (PVs). During the last decade, the prevalence of AF in the UK has increased, and it is projected to grow within the following 30 years [2]. Pulmonary vein isolation (PVI) emerged as the standard of care in symptomatic AF during the last two decades when rhythm control was preferred [3]. Atrial cardiomyopathy is a recently defined condition encompassing changes in the macro and microstructure and various functional aspects of the atria, including the conduit, reservoir, electrical conduction, and contractile function [4,5]. Evidence strongly suggests that atrial cardiomyopathy, which is associated with a heightened risk of AF, is also linked to a greater incidence of AF-related complications such as heart failure, cognitive decline, ischaemic stroke, dementia, and mortality [6]. Notably, the impact of this condition on cognitive and cardiovascular health is distinct from its effects on AF, highlighting its unique prognostic significance. There is an urgent need to develop accurate methods for identifying these issues that can be easily used in a clinical setting. The 12-lead ECG, a conventional clinical tool, may be pivotal in detecting this condition.

The normal P wave, generated by the atria, has various measured parameters, including duration, morphology, voltage, spatial axis, and area. These parameters can be combined to form a P wave index (PWI), such as the morphology–voltage–P wave duration ECG (MVP ECG) risk score. Changes in these parameters, especially in duration and morphology, can indicate atrial chamber enlargement and conduction blocks and are considered risk factors for clinical events such as AF and ischaemic stroke. The predictive value of P wave parameters has been recognised for decades, with an advanced inter-atrial block (IAB) being described in the 1980s as a marker for the risk of AF or atrial flutter [7,8,9]. Furthermore, P wave parameters, including P wave duration (PWD) [10], P wave dispersion (PWDisp) [11], P wave voltage (PWV) [12], P wave terminal force in V1 (PTFV1) [13,14], and P wave area (PWA) [15] have been associated with AF, dementia, stroke, and death. Novel P wave markers were also correlated with AF ablation failure, including beat-to-beat variation, duration-to-amplitude ratio, and a notched P wave [16,17,18]. Modifying the electrical substrate has been suggested to change P wave parameters significantly. Hence, they have been used to predict PVI outcomes, which this study aimed for.

## 2. Material and Methods

### 2.1. Patient Selection and Data Collection

This retrospective observational cohort study included consecutive patients who had completed their first PVI for PAF between January 2018 and December 2019 in Glenfield Hospital, Leicester, UK. Patients taking amiodarone before the procedure were excluded as amiodarone could alter P wave morphology [19]. Patients with previous ablation procedures, patients with pacing devices, patients with valvular disease, patients with additional ablations outside PVs, and patients who did not complete their 12-month follow-up were also excluded. PVI was conducted by contact force radiofrequency ablation, or second-generation catheters were used for cryoballoon ablation. All involved patients had complete PVI with confirmed bidirectional block. Recurrence was defined by AF lasting for 30 s or more on Holter monitoring (24–72 h).

Patient demographics and medication details were obtained electronically by examining clinic appointment letters which provided clinical information, medications, ablation details, and follow-up appointments. Patients who had first-time PVI for PAF had continuous electronic monitoring. P wave parameters were measured before PVI and correlated with procedure success. PVI success was defined by the lack of ECG-documented AF or atrial flutter between 3 months (blanking period) and 12 months following ablation using 12-lead ECG or ambulatory monitoring. The study was reviewed and ethically approved by the University of Leicester ethical committee (reference number: 35479-ia196). The study was reported according to the STROBE guidelines [20].

### 2.2. Ablation Details

In the radiofrequency procedures, a circular mapping catheter was precisely deployed in the superior and inferior PVs, followed by circumferential ablation of the left-sided and right-sided ipsilateral PVs, all meticulously guided by three-dimensional left atrial mapping (CARTO3, Biosense-Webster, Irvine, CA, USA). The PVI was expertly conducted using a 3.5 mm ablation catheter with an externally irrigated tip (ThermoCool SmartTouch Catheter, Biosense-Webster, Diamond Bar, CA, USA), with ablation index guidance. Post-procedure, dormant conduction of the PVs was effectively examined using rapid adenosine triphosphate injection.

In cryoballoon ablation, a seamless transition from a long sheath (8.5 Fr SL0, Abbott Laboratories, Chicago, IL, USA) to a steerable sheath (FlexCath, Medtronic, Minneapolis, MN, USA) set the stage for the insertion of a second-generation cryoballoon (28 mm) into the LA over an inner-lumen circumferential mapping catheter (Achieve, Medtronic, Dublin, Ireland). The cryoballoon was meticulously frozen at the ostium of the superior/inferior left/right PVs. The goal of achieving a bidirectional conduction block between the left atrium and the PVs was pursued as the endpoint of the PVI procedure.

### 2.3. P Wave Analysis

**Figure 1:**
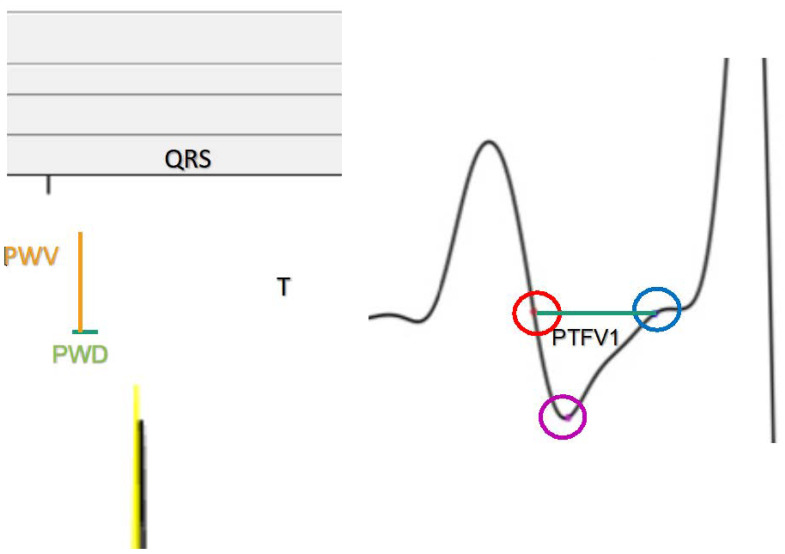
MatLab screenshots demonstrating P wave annotations. PWD: P wave duration; PWV: P wave amplitude; PTFV1: P wave terminal force in V1.

In this study, 12-lead ECG tracings before, during, and after PVI were electronically archived with a resolution of 16 bits. Digital ECGs had a voltage range between 5 and −5 mV (range = 10 mV). The ECG data was filtered using a 1–50 hertz bandpass and a notch filter. One minute of digital ECG tracing directly before PVI and one minute of digital ECG tracing directly after PVI were exported (Figure 1) The ECG data file (1 min) was exported from LabSystems Pro, Massachusetts, USA in the format of .txt files and imported to Matlab for analysis. A digital bandpass filter (second-order Bessel filter) with a cut-off frequency of 1 hertz and 50 hertz was applied. The P wave peak was detected as the peak with a minimum duration/width of 15 ms in the window of interest for P wave peak detection.

The P wave onset detection window was defined from the T wave end to the P wave peak. P wave onset was detected and defined as the point with a minimum perpendicular distance to the line connecting the two T wave and P wave peak points. The MatLab script allows interactive operations, allowing the user to censor and adjust detected points using the computer. This process is demonstrated in Figure 1. Twenty P wave measurements were averaged to one number representing the P wave parameter in that lead. PWD was adjusted similarly to the QT interval adjustment using the Hodges formula. P wave beginning is defined by the first point of rise above the isoelectric line, while P wave peak is defined by the P wave point with the most vertical distance from the isoelectric line. These can be adjusted manually during the measurement process.

The following P wave parameters are produced:

1. PWD: Distance from P wave onset to offset. It represents atrial depolarization;
2. PWV: The area under the P wave was estimated using the trapezoidal method, which involves integrating the total area into a little trapezoid;
3. PWIDisp: The max difference between P wave durations;
4. PTFV1: The product of the maximum absolute amplitude and duration of the second half of the biphasic P wave in mm·s;
5. PWV: Can be calculated by 0.5 × PWD × PWV [7];
6. IAB: Defined by PWD ≥ 120 and a biphasic P wave morphology in leads III, II, or aVF [21].

### 2.4. Statistical Analysis

Categorical variables were expressed as frequency and percentage. The mean ± standard error of the mean was adopted to describe continuous parametric data. Pearson’s χ2 or Fisher’s exact tests were used for categorical variables between groups. Student’s *t*-tests and Mann–Whitney U tests were used to compare continuous variables, including P wave parameters, between the groups depending on the normality of the distribution.

A two-sided *p*-value < 0.05 was considered statistically significant. Statistical analyses were performed using GraphPad Prism V9.3 (San Diego, CA, USA).

### 2.5. Intraobserver Variability Test

There was a human factor in analysing the P wave and manually annotating the P wave’s start and end. Therefore, intraobserver variability tests were conducted to establish the data’s reproducibility. Twenty-two randomly selected 12-lead ECGs were analysed anonymously on two consecutive days. In total, 5280 P waves were analysed and compared twice in two days. Variability was calculated using raw numbers and a percentage. The results of the intraobserver variability showed the highest variability in the PWDisp measurement (4.5 ± 0.3 ms, 19%) followed by PWV (0.03 mV ± 0.001, 13%), PTFV1 (0.4 ± 0.1 mm·s, 10%), PWA (1 ± 0.2 ms·mV, 8%), and PWD (4.5 ± 0.3 ms, 4%).

## 3. Results

### 3.1. Patients Characteristics

After applying the inclusion and exclusion criteria, 211 PAF patients were involved in the final analysis, of which 154 patients (73%) had successful ablation at 12 months, and 81 patients (30%) had radiofrequency ablation. Table 1 demonstrates demographics stratified by procedure outcome. Males comprised 71% of the patients; the mean age was 61 ± 1.3 years. There was no statistically significant difference in age, diabetes mellitus, ischaemic heart disease, cerebrovascular events, hypertension, indexed left atrial volume, body mass index, and the type of antiarrhythmic drugs (ADDs) prescribed between both arms. However, more in the failed group were on long-term AADs (55% versus 25%, *p* < 0.001). The proportion of patients who had radiofrequency ablation and cryoballoon ablation did not differ between successful and failed PVIs.

### 3.2. P Wave Parameters

The PWDc results are demonstrated in Table 2. Increased PWDc was associated with PVI failure in lead I (hazard ratio [HR]: 2.1, 95% confidence interval [CI]: 1.3–4.3, *p* = 0.02), lead II (HR: 1.7, 95% CI: 1.1–3.9, *p* = 0.03), aVL (HR: 4.1, 95% CI: 2.1–7.3, *p* < 0.001), aVF (HR: 1.7, 95% CI: 1.2–4.5, *p* = 0.039), V1 (HR: 1.9, 95% CI: 1.3–4.5, *p* = 0.039), V2 (HR: 2.5, 95% CI: 1.6–5.3, *p* = 0.023), V4 (HR: 2.2, 95% CI: 1.4–5.8, *p* = 0.042), V5 (HR: 2, 95% CI: 1.3–7.4, *p* = 0.034), and V6 (HR: 3.8, 95% CI: 1.9–4.8, *p* < 0.001).

Table 3 demonstrates the PWV results. Lower PWV was associated with failed PVI in lead I (HR: 0.62, 95% CI: 0.22–0.93, *p* = 0.03), lead II (HR: 0.42, 95% CI: 0.13–0.76, *p* = 0.01), aVL (HR: 0.39, 95% CI: 0.24–0.83, *p* = 0.001), and aVF (HR: 0.56, 95% CI: 0.41–0.93, *p* = 0.032).

The PWDisp results in Table 4 did not show a statistically significant difference between both study arms. The PWA results are in Table 5. Lower PWA was associated with failed PVI in lead I (HR: 0.55, 95% CI: 0.21–0.76, *p* = 0.021), lead II (HR: 0.48, 95% CI: 0.34–0.87, *p* = 0.002), aVL (HR: 0.65, 95% CI: 0.45–0.96, *p* = 0.04), and aVF (HR: 0.61, 95% CI: 0.32–0.89, *p* = 0.04). PTFV1 was not statistically different before successful and failed ablation (HR: 1.1, 95% CI: 0.8–1.3, *p* = 0.86). The presence of IAB was associated with failed ablation (HR: 2, 95% CI: 1.4–4.2, *p* = 0.02).

## 4. Discussion

This is the first study to assess PWDc, PWA, PWV, IAB, and PWdisp in all 12 leads before PVI in correlation to the outcome of PAF patients. Furthermore, this is the first study that corrected P wave duration for heart rate to predict PVI outcome. This study proposes three main findings:

1. The PWDc increase in leads I, II, aVL, aVF, V1, V2, V4, V5, and V6 was associated with PVI failure at 12 months;
2. A decrease in PWV and PWA in leads I, II, aVL, and aVF was associated with PVI failure at 12 months;
3. The presence of IAB is correlated with PVI failure.

Recent research has focused on predicting PVI outcomes (Table 6). These generally included increased PWD and PWDisp. Other scoring systems for AF recurrence following AF, including the APPLE (age > 65 years, persistent AF, impaired kidney function, LA diameter ≥ 43 mm, left ventricular ejection fraction < 50% [22]) and MB-LATER scores (male, bundle branch block, LA diameter ≥ 47 mm, type of AF [paroxysmal, persistent, or long-standing persistent]) [23], could not be measured due to some within our PAF cohort without LA diameter (only LA volume) or information about bundle branch block.

Previous studies have assessed P wave parameters to determine whether they can predict new AF incidence [10], as the P wave represents atrial depolarisation and PV cardiac tissue excitation. Furthermore, PWDisp has been utilised to predict persistent AF progression from PAF [24]. With the increased PVI in clinical practice, several studies correlated P wave parameters before and after ablation to clinical outcomes, summarised in Table 7. These mainly included increased PWD, PWDisp, and PTFV1 as predictors of PVI failure. Furthermore, other markers were utilised to predict outcomes. For example, a study demonstrated that the beat-to-beat P wave index had a twofold risk for AF recurrence [17].

In our study, increased *PWDc* was correlated with procedure failure in leads I, II, aVF, aVL, V1, V2, V4, V5, and V6. PWD represents the time for electrical impulses to occur and spread through both atria. Understanding PWD is vital for diagnosing and managing conditions related to atrial activity. Our results are in line with those published by several authors [25,26,27,28,29,30,31,32,33,34,35,36,37]. Furthermore, a meta-analysis containing a total cohort of 1010 patients showed a highly significant association between prolonged PWD and AF recurrence after radiofrequency ablation (Z = 14.20, *p* < 0.000) [38]. High PWDc is seen with failed ablation. This is justified by the higher degree of remodelling and scarring in patients who went on to have failed ablations [39]. The fibrosis causes delayed intra-atrial and inter-atrial conduction, increasing PWD [40,41].

A recent study identified five factors related to ablation failure: female sex, left atrial appendage emptying flow velocity ≤ 31 cm/s, estimated glomerular filtration rate < 65.8 mL/(min·1.73 m^2^), PWD in lead aVF ≥ 120 ms, and P wave duration in lead V1 ≥ 100 ms, and constructed a nomogram [37]. This supports the hypothesis that AF recurrence is influenced by a complex interplay between atrial remodelling and demographics. Although our successful and failed PVIs had PWDc >100 ms and 120 ms in V1 and aVF, respectively, PWDc in failed ablations was significantly more prolonged than in successful ablations, supporting the same hypothesis (HR: 1.7 and 1.9, respectively). Increased PWDc indicates conduction delay signalling and extensive remodelling, which may reduce the effectiveness of ablation in restoring normal sinus rhythm. Also, it contributes to creating re-entrant circuits, wavefront collisions, and forming stable re-entrant pathways that are challenging to eliminate with ablation. This study did not use imaging studies to assess atrial fibrosis, but it would benefit future analysis. It is noted that the significant difference was not notable in some leads, including aVR. This can occur due to the heart’s unique anatomical orientation, the limited projection of the atrial depolarisation vector in this lead, and the baseline characteristics of the P wave in aVR. For example, leads II, III, aVF, and I are particularly relevant for assessing atrial depolarisation vectors and their changes concerning PVI outcomes because they align well with the typical direction of atrial depolarisation.

Decreased *PWV* in leads I, II, AVL, and aVF was associated with PVI failure. Although low-voltage areas signifying LA scarring were mainly detected in persistent AF in the literature, there is evidence that these areas are also seen in PAF [12,42]. These low-voltage areas can cause delayed conduction in the LA, often indicating underlying structural abnormalities such as fibrosis or scarring. These changes can create a more complex and heterogeneous electrical environment that is harder to modify or isolate during ablation. We observed that decreased PWA in leads I, II, AVL, and aVF was associated with PVI failure. A reduced PWA often indicates significant atrial remodelling, fibrosis, and impaired conduction. These factors contribute to less effective atrial depolarisation and could be a sign of advanced atrial disease. Such changes may also indicate a higher chance of ablation failure.

The *PWDisp* was not different between successful and failed PVIs. *PWDisp* is the difference between the maximum and the minimum PWD recorded from the ECG leads. It represents the inhomogeneous propagation of sinus impulses and prolonging inter-atrial and intra-atrial conduction time [43]. Previous studies summarized in Table 7 correlated PWDisp increase with failed PVI. These studies did not comment on the exact ablation technique used. PWDisp provides information about atrial conduction heterogeneity, with greater refractoriness variation and a shorter refractory period leading to AF recurrence [44]. According to a recent study, the PWDisp association with recurrence can also be explained by scar tissue formation identified by electroanatomical mapping [45]. Still, its lack of specificity, sensitivity, and measurement variability limits its predictive value for PVI outcomes. Inconsistent results further contribute to its limited predictive value as it can be affected by multiple factors, including cardiovascular, renal, respiratory, endocrine, and respiratory disorders [43]. Therefore, more comprehensive and integrative approaches are needed to predict PVI success in patients with PAF better using PWDisp.

IAB was associated with an increased risk for AF (HR 3.09, 95% CI 2.51 to 3.79) [46]. Also, in individuals aged 60 to 70 with cardiovascular disease, the 10-year risk of AF was 50% in those with advanced IAB compared with 10% in those with a normal P wave [47].

The *PTFV1* before ablation was not different between successful and failed PVIs. PTFV1 was first described in 1964 [13] and was correlated with the LA volume in 1969 [48]. It represents the negative phase of the P wave in V1. It was considered abnormal when more than 0.03 mm·s [13]. The highest tertile of PTFV1 (78–97 ms) was associated with the highest risk of AF (HR 1.37; 95%, CI 1.23–1.52) and highest risk of stroke (HR 1.13; 95% CI 1.05–1.20) [49]. Also, another study suggested PTFV1 ≥0.06 mm·s was associated with an increased risk of death (HR: 1.76, 95% CI: 1.45–2.12, *p* < 0.001) and AF (HR: 1.91, 95% CI: 1.34–2.73, *p* < 0.001) [50].

PTFV1 was altered after PVI due to the loss of PV antrum signals [51], making it relevant before and after PVI [51]. Regarding the role of PTFV1 in predicting PVI, a previous study correlated PTFV1 < −0.04 mm·s with PVI failure for PAF [29]. Patients with failed PVI in the previous study were older and had larger LA volumes (known to cause abnormal PTFV1 [29]). This can explain the lack of difference in PTFV1 in our study. AF is a complex heart rhythm disorder involving multiple factors, such as electrical, structural, and autonomic changes in the atria. While PTFV1 (a measure of left atrial activation delay) is essential, it does not capture all aspects of AF, like focal triggers, re-entrant circuits, or atrial fibrosis. Additionally, comorbid conditions such as hypertension, diabetes, and other cardiovascular diseases can independently influence the success of PVI and affect PTFV1, making it difficult to assess the relationship between PTFV1 and ablation outcomes. In our study, patients who did not stop their AADs were likely to have failed PVIs. This can be explained by advanced atrial cardiomyopathy or the drugs that differently affect the action potentials that have become heterogeneous and, therefore, lead to the recurrence of AF after ablation [52,53].

This study was not conducted without its limitations. This is a single-centre retrospective study with AF recurrence detected using 12-lead ECG or ambulatory monitoring. Long-term monitoring (implantable loop recorder) was not conducted, and the AF burden was not evaluated. This could have missed subclinical and micro-AF episodes. Imaging studies for assessing LA fibrosis were not utilised. The relatively low sample size was not derived from formal power calculations, increasing the odds of a type 2 error because of the low power. Electroanatomical mapping of the LA was not obtained. Therefore, a correlation between low voltage areas and PWV was not conducted. Flecainide and sotalol used in our cohort could have affected PWD [52,53]. P wave axis and beat-to-beat were not technically possible in this study and are suggested to be performed in future studies.

Patients stopping their antiarrhythmic drugs were included in the analysis. Future studies must match patients with antiarrhythmic drugs and their cessation to limit confounding factors. The Hodges formula is currently not verified as a methodology in the literature to correct PWD for HR. A future dedicated study would help confirm the utility of this formula for future studies utilising PWD.

**Table 6:** Studies that demonstrated factors correlated with ablation failure for atrial fibrillation.

Study	Characteristics Associated with Failed PVI	Comments
Themistoclakis et al., 2008 [54]	Non-paroxysmal atrial fibrillation with duration ↑, hypertension	Longer AF duration (OR 1.03), history of hypertension (OR 1.32), left atrial enlargement (OR 1.55), permanent AF (OR 1.72), and lack of superior vena cava isolation (OR 1.60) were significantly associated with EAT. Independent predictors of LAT were longer AF duration (OR 1.03), history of hypertension (OR 1.65), persistent (OR 2.17) or permanent AF (OR 2.28), and occurrence of EAT (OR 30.62).
Tuan et al., 2010, Vermeersch et al., 2021 [55,56]	Age ↑, 20% of elderly patients had meaningful ↓ QoL over one year	Subjects were divided into three groups according to their age, as follows: Group I: age ≤ 50 (*n* = 141), Group II: age = 51–64 (*n* = 149), and Group III: age ≥65 (*n* = 60). The younger age group had a significantly smaller LA diameter (Group I vs. Group II vs. Group III, 36.89 ± 7.11 vs. 39.16 ± 5.65 vs. 40.77 ± 4.95 mm, *p* = 0.002) and higher LA bipolar voltage (2.09 ± 0.83 vs. 1.73 ± 0.73 vs. 1.86 ± 0.67 mV, respectively, *p* = 0.024), compared with the older AF patients. Vermeersch et al., 2021: global 2 years efficacy of CB-A PVI in persAF is 43.4%. A lower success rate is achieved in the older patients (36.1%) (≥75 years) compared to the younger age group (47.0%)
Chao et al., 2010, Wang et al., 2020, Creta et al., 2020 [57,58,59]	Diabetes mellitus, poor diabetes control	A total of 228 patients with paroxysmal AF who had undergone catheter ablation. Abnormal glucose metabolism (*n* = 65) was defined as diabetes mellitus or an impaired fasting glucose. The AF recurrence rate was also greater in the patients with an abnormal glucose metabolism (18.5% vs. 8.0%, *p* = 0.022) than in those without. Wang et al. 2020: arrhythmia recurrence was significantly higher in the DM group compared to the non-DM group after adjustment for baseline differences (adjusted hazard ratio [HR] 2.24; 95% confidence [CI] 1.42–3.55; *p* = 0.001). Creta et al. 2020: DM was also an independent predictor of AF recurrence on the multivariate analysis (hazard ratio 1.39; 95% confidence interval 95%1.07 to 1.88; *p* = 0.016).
Chang et al., 2011 [60]	Non-paroxysmal atrial fibrillation classification	Very early recurrences of AF occurred in 39 (15%) patients with paroxysmal AF and 26 (34%) with non-paroxysmal AF. Patients with very early recurrence had a higher incidence of non-paroxysmal AF (40% vs. 18.6%, *p* < 0.001), requirement of electrical cardioversion during the procedure, larger left atrial (LA) diameter (43 ± 7 vs. 39 ± 6 mm, *p* < 0.001), lower left ventricular ejection fraction (54 ± 10% vs. 59 ± 7, *p* < 0.001), longer procedural time, and lower LA voltage (1.5 ± 0.7 vs. 1.9 ± 0.8 mV, *p* < 0.001).
Ng et al., 2011a [61]	Obstructive sleep apnoea	Patients with OSA have a 25% greater risk of AF recurrence after catheter ablation than those without OSA (risk ratio 1.25, 95% confidence interval 1.08 to 1.45, *p* = 0.003).
D’Ascenzo et al., 2013 [62]	Recurrence within 30 days, valvular atrial fibrillation	The most powerful predictors of AF ablation failure in the overall population were a recurrence within 30 days (OR 4.30; 2.00–10.80), valvular AF (OR 5.20; 2.22–9.50), and a left atrium diameter of more than 50 mm (OR 5.10 2.00–12.90; all CI 95%).
Letsas et al., 2013 [63]	CHA2DS2-Vasc ≥ 2	
Li et al., 2014a [64]	Chronic kidney disease (PAF patients were at higher risk)	The meta-analysis of these studies showed that CKD was associated with higher AF recurrence rate following single catheter ablation (HR = 1.96, 95% CI 1.35–2.85, *p* = 0.0004). A subgroup analysis showed that CKD has a higher recurrence risk in patients with 100% paroxysmal AF (HR = 2.45, 95% CI 1.28–4.70, *p* = 0.007) than in patients with non-100% paroxysmal AF (HR = 1.65, 95% CI 1.15–2.36, *p* = 0.006).
Qiao et al., 2015 [65]	Alcohol intake ↑	Daily alcohol consumption independently predicted the presence of LVZs (odds ratio [OR], 1.097; 95% confidence interval [CI], 1.001–1.203; *p* = 0.047). During a mean follow-up of 20.9 ± 5.9 months, 40 patients (35.1%) experienced AF recurrence. Success rates were 81.3%, 69.2%, and 35.1% in alcohol abstainers, moderate drinkers, and heavy drinkers, respectively (overall log rank, *p* < 0.001). In conclusion, daily alcohol consumption was associated with atrial remodelling, and heavy drinkers have a substantial risk for AF recurrence after CPVI.
Sultan et al., 2017 [66]	In hospital recurrence, females, non-paroxysmal atrial fibrillation	The multivariate analysis revealed that female sex and AF type prior to the procedure were predictors for AF recurrence. Furthermore, comorbidities such as valvular heart disease and renal failure as well as an early AF relapse were also predictors of AF recurrence during 1 y FU.
Pallisgaard et al., 2017 [67]	Female sex, hypertension, atrial fibrillation duration >2 years, cardioversion < 1 year of ablation	One-year risk of recurrent AF following first-time ablation has almost halved from 2006 to 2014. Hypertension, female sex, cardioversion 1 year prior to ablation, and AF duration for more than 2 years all increased the associated risk of recurrent AF.
Winkle et al., 2017, Pranata et al., 2021 [68,69]	Obesity, body mass index ≥35 kg/m^2^	In patients undergoing AF ablation, increasing BMI is associated with more patient comorbidities and more persistent and long-standing AF. BMI ≥ 35 kg/m^2^ adversely impacts ablation outcomes, and BMI ≥ 40 kg/m^2^ increases minor complications. There was a total of 52,771 patients from 20 studies. Obesity was associated with higher AF recurrence (odds ratio [OR] 1.30 [95% confidence interval [CI] 1.16–1.47], *p* < 0.001; I2: 72.7%) and similar rate of adverse events (OR 1.21 [95% CI 0.87–1.67], *p* = 0.264; I2: 23.9%).
Kuck et al., 2018, Li et al., 2020, Liu et al., 2021 [70,71,72]	Female sex, height in females	After catheter ablation of paroxysmal AF, female sex was associated with an almost 40% increase in the risks of primary efficacy failure and cardiovascular rehospitalization. A total of 689 patients (470 males; age, 53.0 ± 11.7 years) with symptomatic paroxysmal AF receiving index catheter ablation (CA) between 2003 and 2013 were enrolled in this study. Patients in the lower quartiles of height had a lower incidence of AF recurrence (log-rank *p* = 0.022). Height in female patients was strongly associated with AF recurrence (*p* = 0.027) after an index ablation in the 6.33 ± 4.32 years of follow-up.
Kim et al., 2020 [73]	Anaemia before ablation	Non-genetic risk factors for new-onset atrial fibrillation may have a similar impact on different age groups. Except for sex, these non-genetic risk factors can be modifiable.
Chew et al., 2020 [74]	Diagnosis to ablation time ↑	A total of 4950 participants undergoing AF ablation for symptomatic AF. A shorter DAT ≤ 1 year was associated with a lower relative risk of AF recurrence compared with DAT > 1 year (relative risk, 0.73 [95% CI, 0.65–0.82]; *p* < 0.001).
Wu et al., 2021b [75]	Family history of atrial fibrillation	After a mean follow-up of 26.2 ± 19.6 months, 318 out of the 645 patients (49.3%) with FAF and 3339 out of the 7553 patients (44.2%) without FAF experienced AT recurrence, corresponding to annual recurrence rates of 22.8% and 20.2%, respectively. Patients with FAF had a significantly higher risk of AT recurrence (adjusted hazard ratio 1.129, 95% confidence interval 1.005–1.267) in multivariable analysis.
McCready et al., 2011 [76]	Left atrium size ↑	Wide area circumferential ablation with linear and electrogram-based left atrial (LA) ablation was performed in 191 consecutive patients for persistent AF. After a mean follow-up of 13.0 ± 8.9 months, the overall success was 64% requiring a mean of 1.5 procedures. The single procedure success rate was 32%. Left atrial size was a univariate predictor of recurrence after a single procedure (*p* = 0.04). Only LA size [hazard ratio (HR) 1.05/mm with 95% confidential interval (CI) 1.02–1.08] was an independent predictor of recurrence after a single procedure. Only LA size was a univariate predictor of recurrence after multiple procedures (*p* < 0.01).
D’Ascenzo et al., 2013, Bergau et al., 2022 [62,77]	Left atrium diameter ↑	Patients with AF/AT recurrence were older (60 ± 8 vs. 57 ± 10 years; *p* = 0.019), had a higher CHA2DS2-Vasc score (2.47 ± 1.46 vs. 1.98 ± 1.50; *p* = 0.01) and presented with a larger left atrium (LA) diameter (43 ± 5.6 vs. 40 ± 5.1 mm; *p* = 0.002). The LA diameter was also a significant predictor for AF/AT recurrence after CB-PVI (odds ratio: 0.939, 95% confidence interval: [0.886, 0.992], *p* = 0.03).
Platek et al., 2020 [78]	Visfatin ↑ (adipokine made by visceral fat which played a role in inflammation and fibrosis)	Patients with AF recurrence had higher visfatin levels (1.7 ± 2.4 vs. 2.1 ± 1.9 ng/mL; *p* < 0.0001) and multivariate logistic regression analysis containing age, sex, and other independent variables showed that patients with elevated visfatin levels were almost three times more likely to experience AF recurrence (odds ratio 2.92; 95% confidence interval 1.60 to 5.32).
Yunpeng et al., 2020 [79]	Low density lipid ↓, total cholesterol ↓ in females	A total of 71 patients (24.7%) experienced AF recurrence during 3 to 12 months after ablation. By univariate Cox regression survival analysis, TC (HR, 0.63; 95%CI, 0.48–0.82), LDL-C (HR, 0.61; 95%CI, 0.44–0.84), non-paroxysmal AF type (HR, 2.56; 95%CI, 1.52–4.21), and left atrial diameter (HR, 2.18; 95%CI, 1.46–3.24) were significantly associated with AF recurrence.
Reyat et al., 2020 [80]	mRNA plasma PITX2 ↑, mRNA LA PITX2 ↓ (cardiac transcription factor)	Reduced left atrial cardiomyocyte PITX2 and elevated plasma concentrations of the PITX2-repressed, secreted atrial protein BMP10 identify patients at risk of recurrent AF after ablation.
Suehiro et al., 2021 [81]	Intermediate monocytes ↑ (profibrotic marker)	Intermediate monocytes were significantly positively correlated with SRM. PIM ≥ 10% was associated with a VR ≥ 13.3% on LGE-MRI, which predicted AF recurrence after catheter ablation.
Wang et al., 2021a [82]	Carbohydrate antigen-125 ↑	Of the 353 enrolled patients, 85 patients (24.1%) had AF recurrence at the 12-month follow-up. These patients had significantly higher baseline CA-125 levels than those without AF recurrence [(18.71 ± 12.63) vs. (11.27 ± 5.40) U/mL, *p* < 0.001].
Themistoclakis et al., 2008 [54]	Non-paroxysmal atrial fibrillation with duration ↑, hypertension	EAT (within the first 3 months of ablation) developed in 514 (40%) patients and LAT (after 3 months post-ablation) in 292 (22%) patients. Longer AF duration (OR 1.03), history of hypertension (OR 1.32), left atrial enlargement (OR 1.55), permanent AF (OR 1.72), and lack of superior vena cava isolation (OR 1.60) were significantly associated with EAT. Independent predictors of LAT were longer AF duration (OR 1.03), history of hypertension (OR 1.65), persistent (OR 2.17) or permanent AF (OR 2.28), and occurrence of EAT (OR 30.62).
Tuan et al., 2010, Vermeersch et al., 2021 [55,56]	Age ↑, 20% of elderly patients had meaningful ↓ QoL over one year	Subjects were divided into three groups according to their age, as follows: Group I: age ≤ 50 (*n* = 141), Group II: age = 51–64 (*n* = 149), and Group III: age ≥ 65 (*n* = 60). The younger age group had a significantly smaller LA diameter (Group I vs. Group II vs. Group III, 36.89 ± 7.11 vs. 39.16 ± 5.65 vs. 40.77 ± 4.95 mm, *p* = 0.002) and higher LA bipolar voltage (2.09 ± 0.83 vs. 1.73 ± 0.73 vs. 1.86 ± 0.67 mV, respectively, *p* = 0.024), compared with the older AF patients. Vermeersch et al., 2021: global 2-year efficacy of CB-A PVI in persAF is 43.4%. A lower success rate is achieved in the older patients (36.1%) (≥75 years) compared to the younger age group (47.0%)
Chao et al., 2010, Wang et al., 2020, Creta et al., 2020 [57,58,59]	Diabetes mellitus, poor diabetes control	A total of 228 patients with paroxysmal AF who had undergone catheter ablation… abnormal glucose metabolism (*n* = 65) was defined as diabetes mellitus or an impaired fasting glucose. The AF recurrence rate was also greater in the patients with an abnormal glucose metabolism (18.5% vs. 8.0%, *p* = 0.022) than in those without. Wang et al. 2020: arrhythmia recurrence was significantly higher in the DM group compared to the non-DM group after adjustment for baseline differences (HR 2.24; 95% CI: 1.42–3.55; *p* = 0.001). Creta et al. 2020: DM was also an independent predictor of AF recurrence in the multivariate analysis (HR: 1.39; 95% confidence interval 95%1.07 to 1.88; *p* = 0.016).

OSA: obstructive sleep apnoea; DM: diabetes mellitus; AF: atrial fibrillation; HR: hazard ratio; CI: confidence interval; LDL: low density lipid; EAT: early atrial tachyarrhythmia; LAT: late atrial tachyarrhythmia.

**Table 7:** Previous studies that correlated P wave parameters to atrial fibrillation outcomes.

Author and Year	*n*	Recurrence	Cut-Off	Comments
(Jiang et al., 2006) [83]	108	↑ PWDisp		In 108 consecutive patients (93 men, 15 women; mean age 51 +/− 8 years) with paroxysmal AF and no structural heart disease, segmental PVI guided by a Lasso catheter was performed. Forty-one percent (44/108) of AF patients had an early recurrence of AF after a single PVI. Univariate analysis revealed that left atrial diameter (*p* = 0.004), age (*p* = 0.024), and PWDisp (*p* = 0.045) were significantly related to the early recurrence of AF.
(Ogawa et al., 2007a) [26]	27	↑ PWD		At baseline, the maximal P wave duration in patients without AF recurrence (161 +/− 7 ms) was slightly shorter than that in patients with AF recurrence (168 +/− 10 ms, *p* < 0.05). After ablation, patients without recurrence showed a significant reduction of P wave duration from 161 +/− 7 ms to 151 +/− 8 ms (*p* < 0.0001). In contrast, no change of P wave duration was noted in patients with recurrences.
(Okumura et al., 2007) [25]	51	↑ PWD	>150 ms	Fifteen patients suffered from AF recurrences 3 months or more after the PVI. The pre-filtered PWD was significantly longer in patients with recurrence than in those without (166.8 +/− 14.8 ms vs. 145.9 +/− 12.6 ms, *p* < 0.0001).
(Van Beeumen et al., 2010) [27]	39	↑ PWD	≤5 ms change	PWD was significantly shorter in cases of successful outcomes after catheter ablation.
(Caldwell et al., 2013) [28]	100	↑ PWD	>140 ms	The selective cohort consisted of 100 patients out of a total of 170 PVIs: age 58 ± 11 years, 72% male, left ventricular ejection fraction 62 ± 9%, 18% ischaemic heart disease, and 13% diabetic. Thirty-five had prolonged PWD, which was associated with greater AF recurrence rates compared to those without prolonged PWD (63 vs. 38%, *p* < 0.05).
(Salah et al., 2013) [29]	198	↑ PWDisp ↓ PTFV1 ↑PWD	>40 ms ≤ −0.04 mV.ms PWD > 120 ms	PWD ≥ 125 ms, PWDisp ≥ 40 ms, as well as a PTFV1≤ −0.04 mm/sec are good clinical predictors of the already known deleterious sequelae, mainly atrial fibrillation recurrence, post PVI in patients with paroxysmal atrial fibrillation; however, they were not independent from left atrial size and age.
(Blanche et al., 2013) [30]	102	↑PWD	PWD > 140 ms	A filtered PWD >140 ms is a marker of AF recurrence after PVI and probably reflects the extent of atrial remodelling.
(Mugnai et al., 2016a) [31]	426	↑PWDisp ↑PWD		Patients with a prolonged PWD had higher rates of atrial fibrillation recurrence compared with those without prolonged PWD (49 vs. 14%; *p* < 0.001). AF recurrence was significantly associated with prolonged PWD (129 ± 13 vs. 119 ± 11 ms; *p* < 0.001) and P wave dispersion (54 ± 12 vs. 42 ± 10 ms; *p* < 0.001) compared with those who remained in sinus rhythm.
(Hu et al., 2016) [32]	171	↑PWD		PWD variation in lead II is an effective predictor of post-ablation AF recurrence.
(Wu et al., 2016) [84]	204	↑ PWD		During the mean follow-up period of 13.9 ± 6.2 months (range, 3–27 months), 62 patients (30.4%) developed a recurrence of AF. The recurrence rate was higher in patients with advanced IAB than those without advanced IAB (46.3% vs. 26.4%, *p* = 0.006).
(Kanzaki et al., 2016) [85]	76	↑ PTFV1	>9.3 mm·s	During the mean follow-up of 10.2 months, AF recurred in 11 (14%) patients. The PTFV1 month after ablation was significantly higher in the recurrence group compared to that in the non-recurrence group (8.8 ± 3.1 mVms vs. 6.5 ± 2.9 mVms, *p* = 0.017). Higher PTFV1 after cryoballoon ablation was associated with poor prognosis during follow-up. The PTFV1 may be a useful and non-invasive marker to predict the recurrence of AF.
(Jadidi et al., 2018) [33]	72	↑ PWD	>150 ms	PWD ≥150 ms during sinus rhythm measured prior to ablation identifies patients with persistent AF who are at increased risk for arrhythmia recurrence following PVI.
(Knecht et al., 2018) [34]	129	↑ PWD	>120 ms	The recurrence rate was significantly higher in patients with persistent AF, with a higher AF burden, with prolonged PWD, and with an indexed LA volume > 55 mL/m^2^. In multivariable analysis, AFB (hazard ratio: 2.018 (1.383–2.945), *p* > 0.001) and a prolonged P wave (hazard ratio: 2.612 (1.248–5.466), *p* = 0.011) were identified as significant predictors for AF recurrence.
(Yanagisawa et al., 2019) [86]	115	↓ Then ↑ PWD		The reverse dynamics of PWD after initial shortening directly following ablation were significantly associated with PV reconnection.
(Auricchio et al., 2021) [87]	282	↓ PWD	>110 ms	One out of five patients referred for pulmonary vein isolation had a short PWD which was associated with a higher rate of AF after the index procedure. Computer simulations suggest that shortening of atrial action potential duration leading to a faster atrial conduction may be the cause of this clinical observation.
(Supanekar et al., 2021) [88]	160	PR↑ and PWD ↓		Shorter PWD combined with longer atrioventricular node delay, as measured by the proportion of the PR that the P wave occupies, was the best predictor of AF recurrence post-ablation.
(Ohguchi et al., 2021) [35]	84	↑ PWD	≥120 ms	A total of 84 consecutive patients (47 with paroxysmal AF and 37 with persistent AF) who underwent PVI were included. PWD and amplitude in all leads were examined during sinus rhythm immediately after pulmonary vein isolation. During 12 months of follow-up, 20 patients experienced recurrence. The cut-off value of PWD > 120 ms in lead I showed a sensitivity of 75% and specificity of 69% for predicting recurrence. PWD was significantly correlated with left atrial volume, low voltage, and conduction velocity. Significantly higher recurrence rates were observed in patients with PWD > 120 ms than in those with PWD ≤ 120 ms (*p* < 0.001).
(Miao et al., 2022) [36]	273	↑ PWD		In patients with early persistent AF who underwent the radiofrequency ablation procedure for the first time and converted to sinus rhythm, the PWD within 72 h after the procedure was independently associated with the risk of atrial fibrillation recurrence, and the association was linear and positive.
(Huang et al. 2023) [37]	310	PWD ↑ in V1 and aVF	>120 ms aVF, >100 ms V1	Five factors related to ablation failure were as follows: female sex, left atrial appendage emptying flow velocity ≤31 cm/s, estimated glomerular filtration rate <65.8 mL/(min·1.73 m^2^), P wave duration in lead aVF ≥ 120 ms, and that in lead V1 ≥ 100 ms.

PWD: P wave duration; PWdisp: P wave dispersion; PTFV1: P wave terminal force in V1; PVI: pulmonary vein isolation; LA: left atrium; AF: atrial fibrillation.

## 5. Conclusions

The factors associated with PVI failure for PAF include the presence of IAB, increased PWDc, decreased PWV, and decreased PWA, while PWDisp and PTFV1 were not predictive of PVI outcomes.

## Figures and Tables

**Table 1:** Comparison of patient characteristics between successful and failed first-time ablation for paroxysmal atrial fibrillation.

	Total (*n* = 211)	Success (*n* = 154)	Failure (*n* = 57)	*p*-Value
Radiofrequency ablation (%)	81 (38%)	58 (44%)	23 (40%)	0.2
Cryoballoon ablation (%)	130 (62%)	96 (62%)	34 (60%)	0.76
Male (%)	161 (76%)	119 (77%)	42 (74%)	0.47
Age (years)	61 ±1.3	61.1 ± 0.9	61 ± 1.2	0.89
Diabetes mellitus (%)	28 (13%)	21 (14%)	7 (12%)	0.82
Congestive cardiac failure (%)	17 (8%)	12 (8%)	5 (9%)	0.83
Ischaemic heart disease (%)	18 (9%)	13 (8%)	5 (9%)	0.58
Cerebrovascular event (%)	19 (9%)	15 (10%)	4 (7%)	0.42
Hypertension (%)	74 (35%)	54 (35%)	20 (35%)	0.67
Left atrial volume indexed (ml/m^2^)	30 ± 0.7	30 ± 0.4	30.3 ± 0.8	0.71
Body mass index (kg/m^2^)	22.2 ± 1.4	21.8 ± 0.8	23.1 ± 1.6	0.38
Flecainide (%)	59 (28%)	42 (27%)	17 (30%)	0.41
Sotalol (%)	67 (32%)	48 (31%)	19 (33%)	0.5
On flecainide or sotalol long term (%)	72 (34%)	39 (25%)	33 (55%)	**<0.001**
Time flecainide or sotalol stopped (months) after ablation	6.3 ± 0.9	6.3 ± 0.9	6.2 ± 5	0.99

Bold indicates statistical significance.

**Table 2:** Corrected P wave duration (ms) before first-time successful and failed ablation.

	Success (*n* = 154)	Failure (*n* = 57)	HR for Recurrence (95% CI)	*p*-Value
I	132 ± 1.9	145.2 ± 7.2	2.1 (1.3–4.6)	0.02
II	139.1 ± 4.2	149.8 ± 6.3	1.7 (1.1–3.9)	0.03
III	131.1 ± 3	140.5 ± 4.8	1.2 (0.8–1.7)	0.06
AVR	135.5 ± 2.9	140.2 ± 5.3	1.1 (0.7–1.8)	0.33
AVL	121.3 ± 9.2	149.2 ± 8.2	4.1 (2.1–7.30	<0.001
AVF	135.3 ± 4.5	147.8 ± 5.9	1.7 (1.2–4.5)	0.039
V1	135.3 ± 4.7	149 ± 7.1	1.9 (1.3–4.5)	0.029
V2	124.1 ± 4.6	139.5 ± 8.1	2.5 (1.6–5.3)	0.023
V3	128.9 ± 9.8	139.3 ± 4.5	1.3 (0.9–4.1)	0.1
V4	131.3 ± 3.9	145.2 ± 6.9	2.2 (1.4–5.8)	0.042
V5	130.6 ± 4.5	143.4 ± 6.2	2 (1.3–7.4)	0.034
V6	132.1 ± 3	149.3 ± 4.7	3.8 (1.9–4.8)	<0.001

HR: hazard ratio; CI: confidence interval. Bold indicates statistical significance.

**Table 3:** P wave voltage (mV) before first-time successful and failed ablation.

	Success (*n* = 154)	Failure (*n* = 57)	HR for Recurrence (95% CI)	*p*-Value
I	0.24 ± 0.02	0.14 ± 0.02	0.7 (0.53–0.95)	**0.03**
II	0.26 ± 0.02	0.09 ± 0.03	0.45 (0.22–0.65)	**0.009**
III	0.18 ± 0.03	0.1 ± 0.01	0.76 (0.5–1.4)	0.32
|AVR|	0.19 ± 0.03	0.16 ± 0.03	0.96 (0.9–1.1)	0.79
AVL	0.19 ± 0.04	0.05 ± 0.02	0.58 (0.22–0.89)	**0.002**
AVF	0.21 ± 0.01	0.09 ± 0.03	0.67 (0.45–0.87)	**0.023**
V1	0.09 ± 0.04	0.08 ± 0.02	0.98 (0.9–1)	0.87
V2	0.16 ± 0.02	0.15 ± 0.02	1 (0.9–1)	0.87
V3	0.18 ± 0.02	0.12 ± 0.04	0.84 (0.5–1.6)	0.59
V4	0.2 ± 0.02	0.12 ± 0.02	0.82 (0.5–1.8)	0.39
V5	0.17 ± 0.03	0.12 ± 0.02	0.9 (0.6–1.4)	0.48
V6	0.15 ± 0.02	0.11 ± 0.02	0.89 (0.7–1.4)	0.79

HR: hazard ratio; CI: confidence interval. Bold indicates statistical significance.

**Table 4:** P wave dispersion (ms) before first-time successful and failed ablation.

	Success (*n* = 154)	Failure (*n* = 57)	HR for Recurrence (95% CI)	*p*-Value
I	21.2 ± 2.4	24.2 ± 4.6	0.91 (0.64–4.7)	0.72
II	20.3 ± 4.4	23.2 ± 7.5	0.89 (0.78–3.8)	0.59
III	26.4 ± 2.6	23.3 ± 2.7	1.1 (0.42–5.2)	0.66
AVR	22.4 ± 2.7	25 ± 4.4	0.93 (0.48–6.3)	0.79
AVL	42.7 ± 4.2	44.2 ± 3.2	0.96 (0.34–6.2)	0.91
AVF	24.2 ± 4.2	28.4 ± 4.2	0.92 (0.67–3.7)	0.51
V1	25.5 ± 3.2	28.6 ± 3.9	0.89 (0.48–4.4)	0.75
V2	34.3 ± 4.7	33.4 ± 4.9	1 (0.88–1)	0.95
V3	35.2 ± 2.7	37.2 ± 3.3	1 (0.78–1.2)	0.91
V4	34.1 ± 4.6	36.3 ± 4.2	0.99 (0.84–1.3)	0.92
V5	34.2 ± 2.8	37.2 ± 3.8	0.98 (0.81–1.2	0.94
V6	29.1 ± 1.3	34.9 ± 3.2	0.95 (0.72–2)	0.89

HR: hazard ratio; CI: confidence interval. Bold indicates statistical significance.

**Table 5:** P wave area (ms·mV) before first-time successful and failed ablation.

	Success (*n* = 154)	Failure (*n* = 57)	HR for Recurrence (95% CI)	*p*-Value
I	15.8 ± 3.2	10.2 ± 3.3	0.55 (0.21–0.76)	**0.021**
II	18.1 ± 4.4	6.7 ± 0.8	0.48 (0.34–0.87)	**0.002**
III	11.8 ± 2.1	7.0 ± 1.3	0.96 (0.65–2.6)	0.89
|AVR|	12.9 ± 3.4	11.2 ± 2.4	0.99 (0.78–4.2)	0.85
AVL	11.5 ± 1.9	3.7 ± 0.7	0.65 (0.45–0.96)	**0.04**
AVF	14.2 ± 2.2	6.7 ± 0.9	0.61 (0.32–0.89)	**0.04**
V1	6.1 ± 0.9	6.0 ± 1.5	1 (0.9–1)	0.95
V2	9.9 ± 2.4	10.5 ± 0.4	1 (0.9–1)	0.97
V3	11.6 ± 2.1	8.4 ± 1.6	0.95 (0.9–1.2)	0.72
V4	13.1 ± 3.4	8.7 ± 2.7	0.92 (0.87–1.4)	0.69
V5	11.1 ± 1.4	8.6 ± 2.4	0.94 (0.82–1.3)	0.78
V6	9.9 ± 1.1	8.2 ± 1.9	0.9 (0.8–1.4)	0.92

HR: hazard ratio; CI: confidence interval. Bold indicates statistical significance.

## Data Availability

Data relating to this study are available upon reasonable request from the corresponding author.
